# Bacterial respiratory inhibition triggers dispersal of *Pseudomonas aeruginosa* biofilms

**DOI:** 10.1128/aem.01101-23

**Published:** 2023-09-20

**Authors:** Anna C. Zemke, Emily J. D'Amico, Angela M. Torres, Grace P. Carreno-Florez, Patrick Keeley, Matt DuPont, Naomi Kasturiarachi, Jennifer M. Bomberger

**Affiliations:** 1 Division of Pulmonary, Allergy and Critical Care Medicine, Department of Medicine, University of Pittsburgh, Pittsburgh, Pennsylvania, USA; 2 Department of Microbiology and Molecular Genetics, University of Pittsburgh, Pittsburgh, Pennsylvania, USA; Danmarks Tekniske Universitet, Lyngby-Taarbæk, Denmark

**Keywords:** *Pseudomonas*, biofilm, dispersal, respiration, cyclic-di-GMP

## Abstract

*Pseudomonas aeruginosa* grows as a biofilm under many environmental conditions, and the bacterium can disperse from biofilms via highly regulated, dynamic processes. However, physiologic triggers of biofilm dispersal remain poorly understood. Based on prior literature describing dispersal triggered by forms of starvation, we tested bacterial respiratory inhibitors for biofilm dispersal in two models resembling chronic airway infections. Our underlying hypothesis was that respiratory inhibitors could serve as a model for the downstream effects of starvation. We used two experimental conditions. In the first condition, biofilms were grown and dispersed from the surface of airway epithelial cells, and the second condition was a model where biofilms were grown on glass in cell culture media supplemented with host-relevant iron sources. In both biofilm models, the respiratory inhibitors potassium cyanide and sodium azide each triggered biofilm dispersal. We hypothesized that cyanide-induced dispersal was due to respiratory inhibition rather than signaling via an alternative mechanism, and, indeed, if respiration was supported by overexpression of cyanide-insensitive oxidase, dispersal was prevented. Dispersal required the activity of the cyclic-di-GMP regulated protease LapG, reinforcing the role of matrix degradation in dispersal. Finally, we examined the roles of individual phosphodiesterases, previously implicated in dispersal to specific triggers, and found signaling to be highly redundant. Combined deletion of the phosphodiesterases *dipA*, *bifA*, and *rbdA* was required to attenuate the dispersal phenotype. In summary, this work adds insight into the physiology of biofilm dispersal under environmental conditions in which bacterial respiration is abruptly limited.

**IMPORTANCE:**

The bacterium *Pseudomonas aeruginosa* grows in biofilm communities that are very difficult to treat in human infections. Growing as a biofilm can protect bacteria from antibiotics and the immune system. Bacteria can leave a biofilm through a process called “dispersal.” Dispersed bacteria seed new growth areas and are more susceptible to killing by antibiotics. The triggers for biofilm dispersal are not well understood, and if we understood dispersal better it might lead to the development of new treatments for infection. In this paper, we find that inhibiting *P. aeurginosa’s* ability to respire (generate energy) can trigger dispersal from a biofilm grown in association with human respiratory epithelial cells in culture. The dispersal process requires a protease which is previously known to degrade the biofilm matrix. These findings give us a better understanding of how the biofilm dispersal process works so that future research can discover better ways of clearing bacteria growing in biofilms.

## INTRODUCTION

In chronic *Pseudomonas aeruginosa* airway infections, such as those found in cystic fibrosis, bacteria grow as biofilms on the epithelial surface or as suspended aggregates in the viscous airway secretions (1, 2). Aggregate communal growth protects bacteria from killing by antibiotics and makes clearance by the host immune system more difficult (3). In response to environmental triggers, bacteria growing in biofilms can disperse and go on to seed biofilms in new locations (4, 5). Biofilm dispersal is a highly regulated, energy-dependent process that requires degradation of the extracellular matrix components such as polysaccharides or DNA (6, 7). A better understanding of biofilm dispersal physiology may lead to improved treatments for acute and chronic infections, as this lifestyle switch directly affects virulence and antibiotic tolerance (8, 9).

The lifestyle switch between sessile biofilm and motile growth is mainly regulated by the second messenger cyclic-di-GMP (4). Triggers of biofilm dispersal include nitric oxide (NO), oxygen deprivation, carbon source starvation, pyruvate depletion, abrupt changes in temperature, and a number of toxic substances including mercury (10–14). Exposure to these substances leads to a drop in cyclic di-GMP-concentration, often within minutes, and resumption of a motile lifestyle (15). NO is the most studied dispersal trigger for *P. aeruginosa*, with wide concentrations of NO donors having been reported in the dispersal literature (16–18). At low concentrations, NO functions as a signaling molecule, however high concentrations of NO are very toxic to *P. aeruginosa* and inhibit respiration (19). Because respiratory chain inhibition might serve as another form of physiologic starvation, we hypothesized that respiratory arrest might also trigger biofilm dispersal.

*P. aeruginosa* has a highly branched aerobic respiratory chain, as well as an anaerobic respiratory chain, that can support vigorous growth (20). Additionally, under certain growth conditions, the organism produces high concentrations of cyanide anions while sustaining its own growth (21). Cyanide production acts a toxin for neighboring organisms, and *P. aeruginosa* can protect itself during cyanogenesis through the expression of a cyanide-insensitive terminal oxidase (22). We took experimental advantage of this unique biology to test the hypothesis that respiratory inhibition triggers *P. aeruginosa* biofilm dispersal.

## MATERIALS AND METHODS

### Reagents

Reagents were purchased from Sigma Chemical, including potassium cyanide (KCN), sodium azide, rotenone, paraquat, N-acetyl cysteine, sodium hypochlorite, and carbonyl cyanide 3-chlorophenylhydrazone (CCCP). Agents were used at the following concentrations: potassium cyanide, 17–300 μM; sodium azide, 0.125–5 mM; CCCP, 40 µM; rotenone, 350 µM; paraquat, 1 mg/mL, sodium hypochlorite, 2 µg/mL estimated free chlorine, and N-acetyl-cysteine, 10mM.

### Strains and growth conditions

Overnight cultures were routinely grown in lysogeny broth (LB; Sigma) at 37°C on a roller drum. Strains constructed and primers used are listed in Table S1. Deletion strains were created using the homologous recombination protocol published by Hmelo (23). Single deletion strains of *dipA* and *rbdA* were previously published in (17). Compound deletion strains were constructed using homologous recombination as described in references (17, 23). To confirm final strain identity, whole-genome sequencing was performed on a NextSeq 500 System (Illumina) using 2 × 150 bp libraries at the University of Pittsburgh’s Microbial Genome Sequencing Center. Breseq version 0.28.1 was used for variant calling with alignment to the PAO1 genome (24). No additional mutations were found in the strains used.

To create the strain that constitutively expresses *cioAB*, the open reading frame of *cioAB* was inserted into pUC18-mini-Tn7 that contained the constitutive promoter for the gene *ntp2* upstream of the multiple cloning site. This vector allows for genomic insertion at a single, known *att*Tn7 site in *P. aeruginosa*. The construct was then transferred into PAO1 through mating and insertion at the correct site was confirmed by PCR (25). Complementation of *lapGD* was done by inserting the operon and 500 bp upstream into the *att*Tn7 site in the PAO1 ∆*lapGD* strain. The operon was amplified by PCR from PA14, restriction digested and cloned using ligation into pUC18-mini-Tn7 and inserted into the deletion strain as described above. The entire insert was sequence confirmed to exclude point mutations.

### Model for dispersal of biofilms from an abiotic surface

Experiments were done as described in Zemke et al. (17) without modification until the dispersal step, at which point alternative agents were used. Briefly, biofilms were grown on glass coverslips under static conditions in minimal essential medium (MEM) supplemented with 62.5 µM holotransferrin and 31.25 µM hemoglobin for either 6 or 24 h. An initial inoculum of 2.25 × 10^6^ bacteria in 250 µL of medium for each 100 mm diameter coverslip was used. After incubation, KCN was added to the dish to induce dispersal and the dish was incubated for another 15 min. After dispersal, specimens were fixed and imaged. Note that keeping the control and cyanide treatment groups in separate plastic bags within the incubator prevented inadvertent dispersal of the controls by gaseous cyanide released during the assay.

### Biofilm imaging

Biofilms were grown on glass and dispersed as described above. Cultures were imaged with a widefield inverted NikonTi microscope at 40×. For quantification, at least six random z-stack fields were captured, the z-stack was then trimmed to include only images with intact bacteria present, z-stacks had a threshold applied and the volume was determined using Nikon Elements v4.30.02 software. For representative images in the manuscript, images were deconvoluted using identical settings across all images and volumetric projections were rendered using Nikon Elements software.

### Human cell culture and biotic biofilm dispersal model

The experiments were performed as described in Zemke et al. which discusses model development in detail (17). CFBE41o- immortalized human bronchial epithelial cells (AECs) were grown at the air-liquid interface on 12 mM Transwell filter inserts (Corning). The filters were inoculated with rinsed, diluted overnight cultures. Bacteria were allowed to attach for 1 h, with a mean of 5 × 10^4^ bacteria attached, the apical supernatant was changed and the co-culture was incubated for 5 h in a cell culture incubator at 37°C with 5% CO_2_ under static conditions (total incubation time of 6 h). Note that for cyanide dispersal, control and treatment filters needed to be placed in separate plastic bags within the incubator to prevent the dispersal of the control samples by gaseous cyanide. For dispersal, pre-warmed media containing the dispersal agents was placed on the apical surface of the co-culture for 15 min, at which point the dispersed bacteria were counted by serial dilution. To determine the number of bacteria in biofilms grown on AECs, the adherent biofilm bacteria were removed with 0.1% Triton X-100 and counted by serial dilution as previously described (17).

### Statistics

At least three replicates were performed for all experiments, and typically five to eight replicates were completed. Statistical analysis was done in PRISM 9.0 software (GraphPad, San Diego, CA, USA). Data are displayed as mean ± standard deviation. CFU counts were log-transformed, and then either a *t* test or one-way analysis of variance (ANOVA) was done, depending on experimental design.

## RESULTS

### Inhibition of respiration by cyanide and azide trigger biofilm dispersal

Nitric oxide, the most studied biofilm dispersal cue in *P. aeruginosa*, is both a respiratory inhibitor and a gaseous signaling molecule, leading us to probe whether other respiratory inhibitors can trigger biofilm dispersal. Because biofilm lifestyle growth is a therapeutic problem in chronic airway disease, and bacterial behavior can vary widely depending on growth conditions, we used two models that recapitulate specific aspects of the human airway. In the first model, biofilms are grown on a glass substrate, but in minimal essential medium (a common mammalian cell culture medium) that is supplemented with the most abundant iron sources in the airway: holotransferrin and hemoglobin. In the second model, biofilms are grown on the surface of polarized airway epithelial cells. The models differ in the nutritional environment for biofilm growth and have different surface substratum characteristics (smooth glass as compared to the apical epithelial surface that is highly decorated with mucins). Both models were previously validated using nitric oxide donors, as these are the best understood dispersal agents. In the airway epithelial co-culture model, exposure to nitric oxide donors lead to a ~90% reduction in biomass and ~1 log increase in dispersed bacteria within 15 min (17). We chose a 15-min time point because it is sufficient for a dispersal response in other models (26, 27), long enough to be experimentally repeatable, minimized the potential for bacterial growth to confound the results, and has been previously published by our group (17).

**Fig 1:**
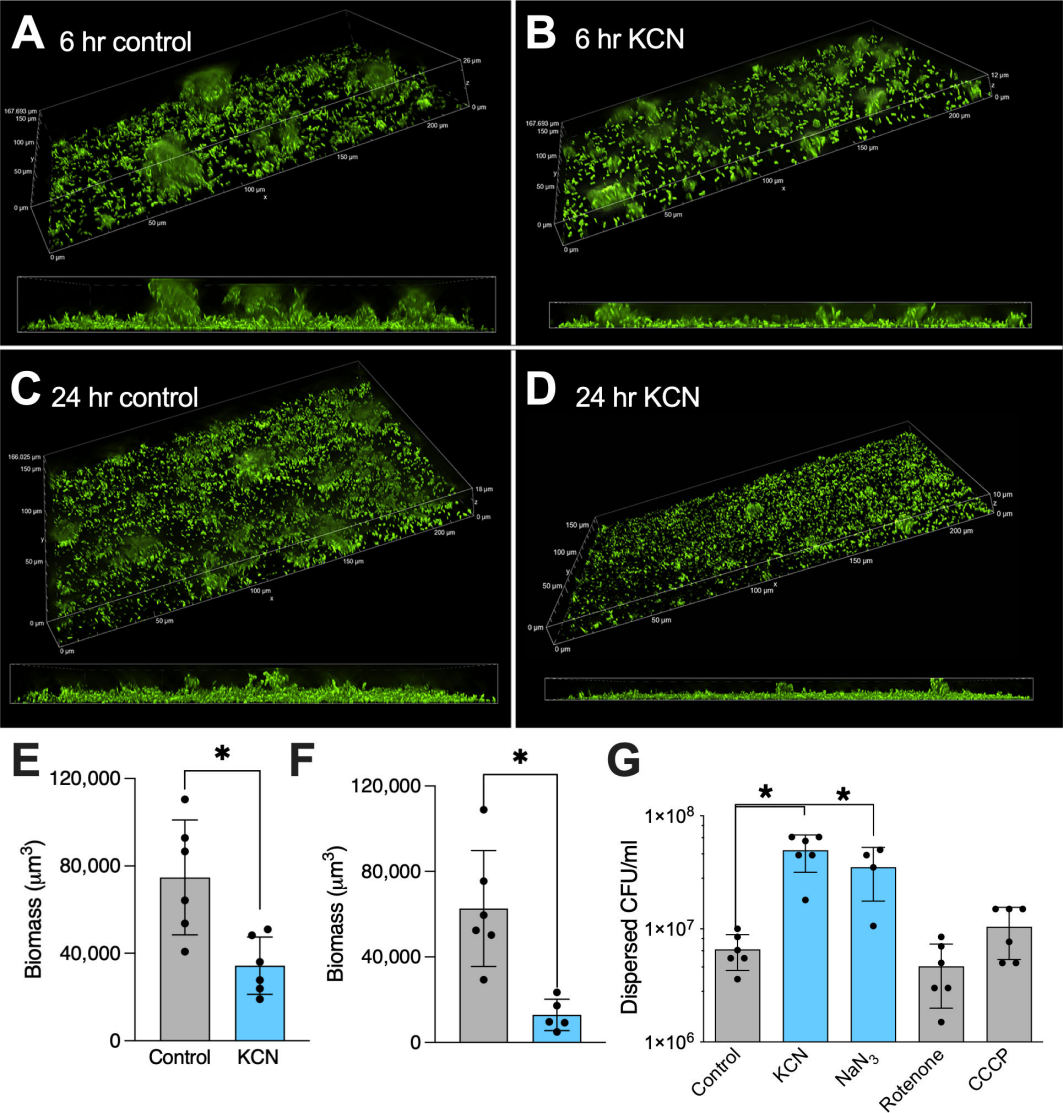
Cyanide and azide disperse *P. aeruginosa* biofilms. (**A–F**) PAO1-gfp biofilms were grown in iron supplemented medium on a glass surface and treated with control medium or 300 µM KCN for 15 min and then fixed for imaging. All imaging was done at 40× magnification. The *x* projections are 224 µM in length, and z-stacks range from 10 to 26 μM in displayed images. (**A**) Representative image of 6 h control biofilm. (**B**) Representative image of 6 h biofilm dispersed with KCN. (**C**) Representative image of 24 h control biofilm. (**D**) Representative image of 24 h biofilm treated with KCN for 15 min. (**E**) Biomass quantification for 6 h biofilms, *P* < 0.05 by unpaired *t* test. (**F**) Biomass quantification of 24 h biofilms, *P* < 0.05 by unpaired *t* test. (**G**) Biotic PAO1 biofilms were grown on CFBE41o- airway epithelial cells and treated for 15 min with the indicated compounds. Dispersed bacteria were counted by serial dilution. *P < 0.001 by one-way ANOVA followed by Dunnett’s multiple comparison test for KCN and NaN_3_.

First, we tested if potassium cyanide triggered biofilm formation in *P. aeruginosa* strain PAO1 biofilms grown on a glass surface, as described previously (17). With 15 min of exposure to cyanide, the imaged remaining biomass decreased to a mean of 43% of the control (Fig. 1A and B, *P* = 0.007 by unpaired *t* test, shown in Fig. 1E). Similar results were obtained with biofilms grown for 24 h prior to treatment (Fig. 1C and D, *P* = 0.007 by unpaired *t* test, shown in Fig. 1F).

**Fig 2:**
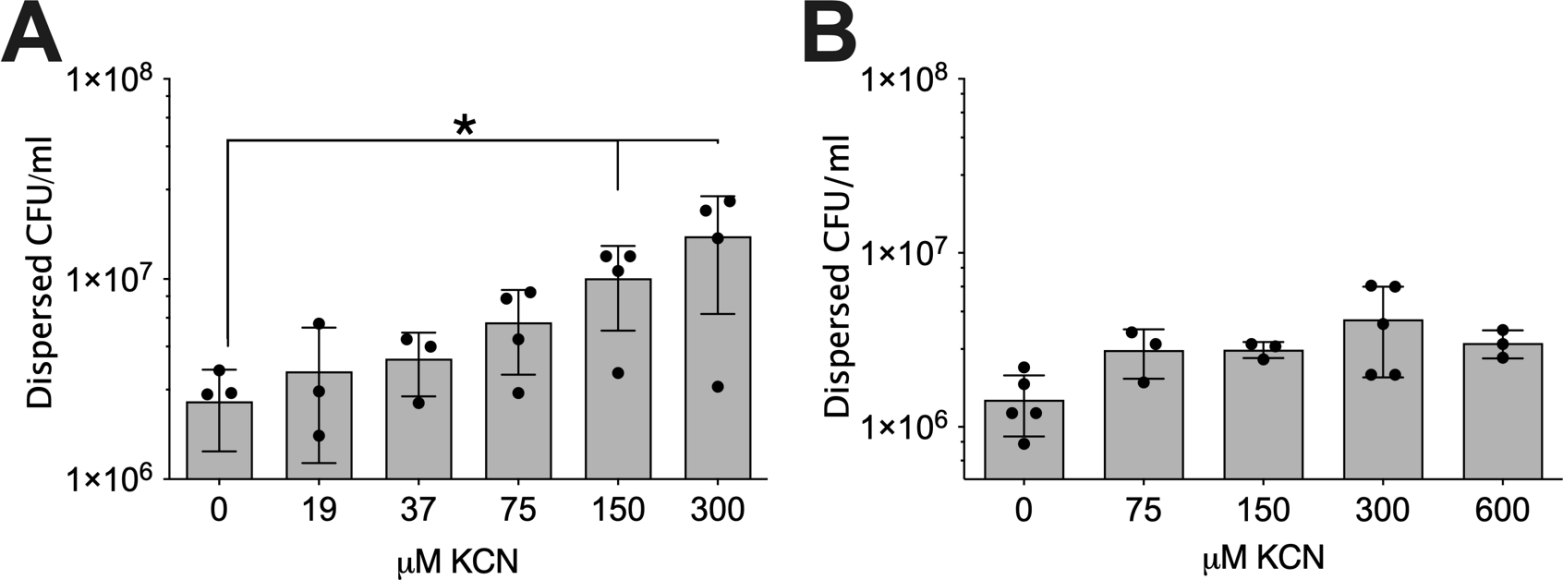
CioAB attenuates cyanide-mediated dispersal. (**A**) Dose-response curve for KCN dispersal of PAO1 biofilms grown on AECs. (**B**) Dose-response curve of dispersal by KCN in PAO1 compared to a strain constitutively overexpressing *cioAB*. When data from (**A**) and (**B**) are analyzed together, a genotype effect present by two-way ANOVA, *P* < 0.05.

Next, we tested multiple potential respiratory inhibitors in a biofilm model in which PAO1 was grown in association with the apical surface of CFBE41o- airway epithelial cells, here after referred to as “biotic biofilms” in reference to the growth substrate (Fig. 1G) (17, 19, 28–30). Both 300 µM KCN and 5 mM sodium azide caused a 1-log increase in the number of dispersed bacteria within 15 min, while rotenone and CCCP did not (Fig. 1A). We chose concentrations of dispersal agents that were well above those needed to inhibit enzymatic activity of their targets to minimize experimental variability, and in the case of cyanide specifically, chose the concentration where the dispersal response plateaued (see Fig. 2A). Cyanide and azide anions both bind heme and inhibit enzymatic activity of a wide range of metalloenzymes, including cytochrome a3 oxidase activity (31). *P. aeruginosa* has a branched respiratory chain at the NADH dehydrogenase level, and NDH-1 (which is homologous to Complex 1 in eukaryotes and inhibited by rotenone) is not required for aerobic growth (32, 33), thus rotenone only inhibits *P. aeruginosa* growth under anaerobic conditions. Flagellar motility (and thus proton motive force) is required for active dispersal (11, 17). A proton ionophore such as CCCP would not be predicted to cause dispersal and has previously been used to differentiate active dispersal from mechanical disruption (11). Taken together, these data show that exposure to cyanide leads to biofilm dispersal—both in biofilms grown on airway epithelial cells and those grown on a glass surface.

Respiratory inhibition might also lead to the production of reactive oxygen species; thus, we tested if two oxidants (paraquat and sodium hypochlorite) caused dispersal of biofilms grown on epithelial cells (Fig. S1) and if the addition of N-acetyl cysteine would prevent dispersal by cyanide (**Fig. S1**). Oxidant exposure did not trigger dispersal and the addition of a ROS scavenger did not prevent dispersal. Prior work by others suggests that oxidant stress may lead to increased biofilm formation via signaling through the diguanylyl cyclase PA3177 (34).

### Dispersal by cyanide is attenuated by expression of cyanide-insensitive oxidase

Cyanide anions bind to heme, and thus, it is possible that the cyanide is directly interacting with a heme-containing metalloprotein sensor rather than triggering dispersal through respiratory inhibition. *P. aeruginosa* conditionally expresses a cyanide-insensitive terminal oxidase, CioAB, which is induced by exposure to low concentrations of cyanide (35). We hypothesized that constitutive expression of *cioAB*(PA3929–PA3930) would protect from cyanide dispersal through supporting respiration. A dose-dependent increase in dispersed bacteria was seen in PAO1 biotic biofilms grown on airway epithelial cells, with the maximum effect at 300 µM (Fig. 2A). A strain that constitutively overexpressed *cioAB* did not demonstrate dispersal when treated with the KCN up to 600 µM (Fig. 2B). These results suggest that dispersal is induced in response to abrupt respiratory inhibition (i.e., cyanide exposure), and that preservation of respiration prevents biofilm dispersal.

### Dispersal requires the matrix-degrading protease LapG

**Fig 3:**
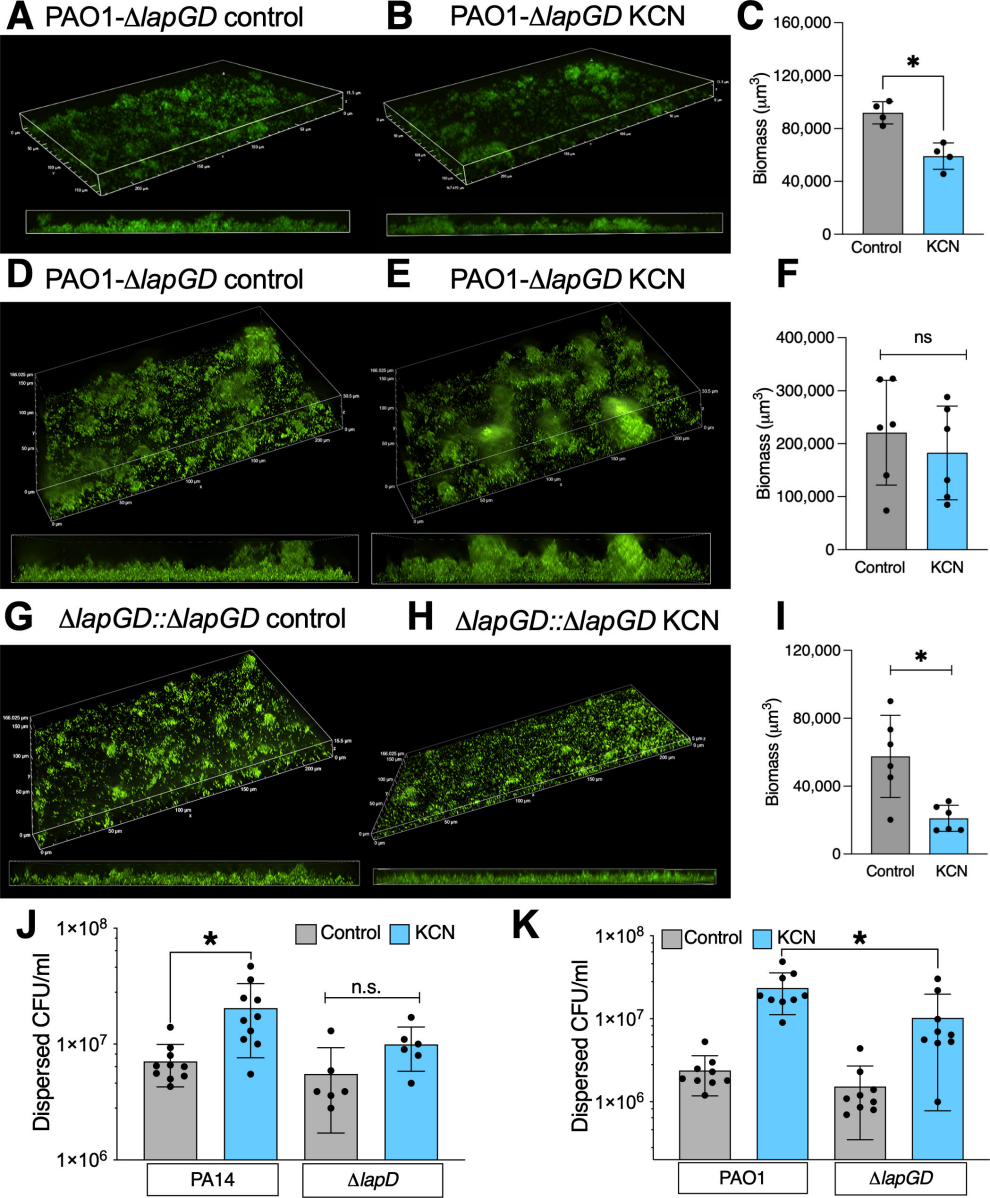
LapGD mediates cyanide induced biofilm dispersal. (**A–I**) PAO1-gfp biofilms were grown in iron supplemented medium on a glass surface and treated with control medium or 300 µM KCN for 15 min and then fixed for imaging. All imaging was done at 40× magnification. The *x* projections are 224 µM in length, and z-stacks range from 18 to 24 μM in displayed images. (**A**) Representative image of *∆lapGD* biofilm grown for 6 h. (**B**) Representative image of ∆*lapGD* biofilm grown for 6 h and dispersed with KCN. (**C**) Quantification of ∆*lapGD* 6 hr biofilms dispersed with control media or KCN. (**D**) Representative ∆*lapGD* bioflm grown for 24 h. (**D**) Representative image of *∆lapGD* biofilm grown for 24 h and dispersed with KCN. (**F**) Quantification of *∆lapGD* 24 h biofilms dispersed with control media or KCN. (**G**) Representative image of PAO1 ∆*lapGD::lapGD* biofilm grown for 24 h. (**H**) Representative image of PAO1 ∆*lapGD::lapGD* biofilm grown for 24 h and dispersed with KCN. (**I**) Quantification of ∆*lapGD::lapGD* 24 h biofilms dispersed with control media or KCN. (**J–K**) Biofilms of PA14 (J) or PAO1 (K) and isogenic deletions of *lapGD* were grown on CFBE41o- airway epithelial cells and dispersed with 300 µM KCN for 15 min. Dispersed bacteria were counted by serial dilution. *P < 0.05 by one-way ANOVA followed by Dunnett post hoc testing.

Cyclic-di-GMP is a second messenger that controls lifestyle switches between motile and attached growth in *P. aeruginosa*. Concentrations of the second messenger are regulated by phosphodiesterase (PDEs) and diguanylylcyclase (DGC) enzymes, and specific enzymes are required for dispersal in response to specific triggers (10, 18, 36). Therefore, we screened the in-frame deletion library published by Ha et al. in the biotic biofilm model to determine which proteins were required for cyanide-induced biofilm dispersal (37). From the library, only deletion of *lapD* decreased biofilm dispersal (screen data shown in Table S2, methods in Supplementary Methods). While included in the library of putative PDE and DGC enzymes, LapD (encoded by PA1433) is a cyclic-di-GMP regulated effector that binds the protease LapG (encoded by PA1434), which cleaves the matrix component CdrA, encoded by PA4625 (38, 39). When biofilms were grown on glass for 6 h, biofilm biomass was not significantly different between parental and ∆*lapGD* strains (Fig. 1A and Fig. 3A). Dispersal with cyanide was seen, but the effect size was smaller (Fig. 3A through C, *P* < 0.05 by unpaired *t* test). When growth time was extended to 24 h, biomass was three times higher in the ∆*lapGD* strains as compared to parental (Fig. 1C and 3D), and dispersal with KCN was lost (Fig. 3D through F, *P* < 0.05 by unpaired *t* test). Complementation of the ∆*lapGD* strain with a single copy, chromosomal insertion of the operon and the 500 bp upstream returned biofilm formation to the parental magnitude and restored dispersal at 24 h (Fig. 3G through I). In PA14, loss of *lapD* prevented dispersal of biotic biofilms from the surface of airway epithelial cells by cyanide (Fig. 3J). In PAO1, deletion of the *lapGD* operon significantly decreased dispersal of biotic biofilms (Fig. 3K) (39). These data suggest that cyanide biofilm dispersal requires the canonical step of matrix degradation, as would be predicted from the literature (39).

### Functional redundancy of phosphodiesterases in dispersal

**Fig 4:**
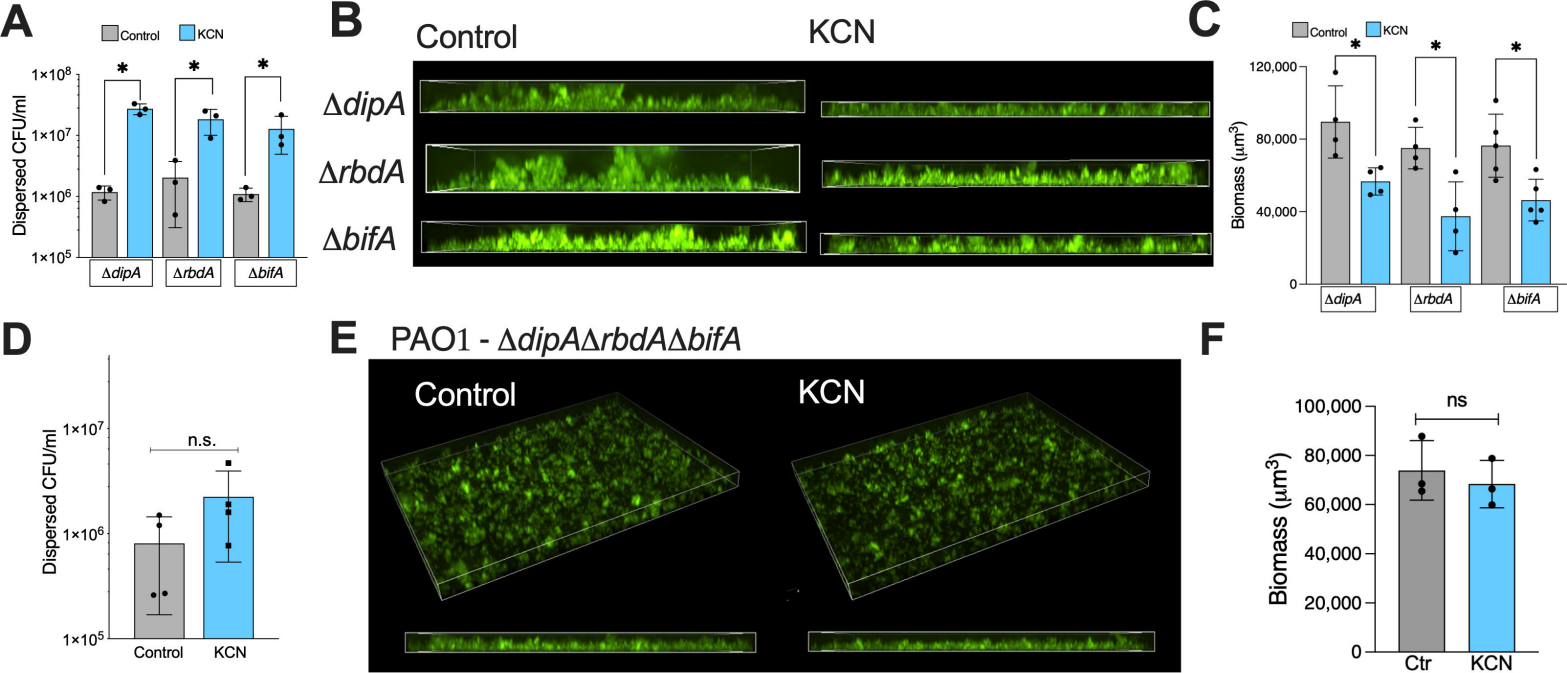
Deletion of multiple PDEs required to inhibit dispersal. (**A**) Biofilms of single deletion strains were grown on CFBE41o- airway epithelial cells and dispersed with 300 µM KCN for 15 min. Dispersed bacteria were counted by serial dilution. (**B and C**) Biofilms of indicated strains were grown on glass in MEM-Fe for 6 h and treated with control solution or cyanide. Representative maximum intensity projection x-z stacks shown or indicated strains and treatments shown in (**B**) and quantification is shown in (**C**). (**D**) Biofilms of PAO1-gfp ∆*bifA*∆*rbdA*∆*dipA* were grown on CFBE41o- cells and dispersed with cyanide as above. (**E**) Representative images of triple deletion strain biofilm grown on glass and dispersed with cyanide. (**F**) Quantification of data shown in (**E**). *P < 0.05 in one-way ANOVA followed by Sidak’s test for panels (A and C). Unpaired *t* tests used for panels (D and F). * indicates *P* < 0.05.

In our screen, we were surprised that deletion of the individual PDEs *dipA*(PA5017)*, bifA*(PA4367), and *rbdA*(PA0861) did not block dispersal, because these enzymes have been shown to be required for dispersal in response to nitric oxide (DipA), oxygen deprivation (RbdA), or a broad range of triggers in the literature (DipA, BifA) (36, 40, 41). Because of these unexpected results in the screen, we formally tested the strains in both the biotic and abiotic models (Fig. 4). Individual deletion of *rbdA*, *dipA*, and *bifA* did not change the magnitude of dispersal by cyanide in biofilms grown in association with CFBE41o- airway epithelial cells (Fig. 4A). In fact, the Δ*dipA* strain showed a statistically significant increase in fold-change CFU dispersed as compared to the parental strain (*P* = 0.0097 by one-way ANOVA). All strains had similar biofilm, as measured by CFU in the assay (Fig. S2). When these strains were grown on glass, dispersal was observed for all strains (representative images in Fig. 4B and quantification in Fig. 4C). We hypothesized that dispersal signaling in these models might be redundant at the individual PDE level, but still be regulated via cyclic-di-GMP. Deletions of any two of these genes did not block dispersal, although we saw progressive attenuation in the magnitude of dispersion with these compound deletion strains (Fig. S3). The triple deletion stain Δ*bifA*Δ*dipA*Δ*dipA* did not disperse in response to cyanide when grown on either glass or airway epithelial cells (Fig. 4D through F). Taken together, these data suggest redundancy in the PDEs required to mediate cyanide-induced biofilm dispersal.

## DISCUSSION

The switch from sessile to planktonic growth is a fundamental physiologic decision for bacteria that has wide-reaching implications regarding the ability of an organism to seed new locations, vulnerability to antibiotics, immune clearance, toxins, and predation, as well as multiple potential applications in human disease. For example, in cystic fibrosis, *P. aeruginosa* infects the airways and grows in high cyclic-di-GMP communities encased in matrix (biofilms) that have extraordinary tolerance to antibiotics. If we were able to trigger bacterial dispersal from airway biofilms, it might improve the ability of antibiotics to clear these chronic infections. However, to date the physiologic or environmental cues that lead to dispersal remain poorly understood. In this work, we identified bacterial respiratory inhibition as a trigger for *P. aeruginosa* biofilm dispersal. Dispersal partially required expression of the matrix-degrading enzyme LapG, and is also cyclicdi-GMP signaling dependent, although the details of this regulation remain incompletely understood.

Cyanide is a potent toxin produced by *P. aeruginosa*, with production regulated by quorum sensing. Concentrations of cyanide in CF sputum may reach 150 µM, which is sufficient to decrease epithelial ciliary function (42, 43), and over longer exposures might result in epithelial cell death. In interbacterial competition, cyanide production gives *P. aeruginosa*a metabolic advantage over competitors such as *Burkholderia* spp. and *Staphylococcus aureus* (44). Within a *P. aeruginosa* population, cyanide is one factor that polices cheaters that do not produce metabolically expensive quorum sensing-regulated common goods (45). Given the potential for self-intoxication, the importance of cyanide in maintenance of energetically expensive quorum sensing within the population, and the importance of cyanide production in virulence, it is unsurprising that cyanide production and defenses are tightly regulated. While a useful tool for studying bacterial physiology, any translation is limited by the obvious host toxicity of both cyanide and azide. Also, while these data strongly support the inhibition of respiration as the mechanism, we cannot exclude the concurrent possibility that cyanide is signaling through additional metalloproteins.

It is perhaps unsurprising that respiratory inhibition would also trigger biofilm dispersal as the absence of a terminal electron acceptor or perhaps the presence of a respiratory inhibitor both represent an existential threat to a biofilm community by impairing bacterial metabolism and energy-dependent processes like cell replication. There is precedent for a redox state to determine exopolysaccharide matrix production, another process tightly regulated by cyclic-di-GMP (46). Recently, the PDEs RmcA and MorA were shown to be needed to downregulate EPS production in the setting of nutrient starvation of mature biofilms (47). While not clearly cyclic-di-GMP dependent, the stringent response triggers dispersal in *P. putida* (48), an additional example of the potential of links between metabolic state(s) and the switch to a motile lifestyle. The mechanistic link between energy status, redox state, or nutrient levels and cyclic-di-GMP signaling remains to be determined in the current system, and likely varies with the nutritional environment.

Dispersal in the model at least partially requires the activity of the protease LapG. LapG is regulated by the cyclic-di-GMP binding protein LapD, via sequestration of LapG by LapD when concentrations of cyclic-di-GMP are high, leading to a decrease in protease activity (30). One target of LapG is the adhesin CdrA, which binds the polysaccharide matrix components pel and psl (39, 49, 50). CdrA can be cleaved by LapG, as well as LasB, a secreted protease and be released into the environment, resulting in a freeing of the matrix it tethers (51). In biofilms grown on glass, *lapGD* was required for cyanide-mediated dispersal at 24 h, with dispersal being present but with a smaller effect size at 6 h. Also notable in our data is the threefold larger biomass seen at 24 h in the LapGD, as opposed to parental strain, which suggests there may be ongoing biofilm remodeling or spontaneous dispersal in our model by the 24-h time point. While the adhesin that is cleaved differs between species, LapG is required for carbon starvation-induced dispersal in *P. fluorescens*, *P. putida*, and *P. aeruginosa*; thus, it is not surprising that LapG influences dispersal in our models (14).

Regulation of cyclic-di-GMP phenotypes is highly dependent on the environmental context of the phenotype, with the role of any specific enzyme varying with the precise growth conditions (17, 52, 53). However, it is well established that dispersal is accompanied by a drop in cyclic-di-GMP due to increased hydrolysis by PDEs. There are at least 14 enzymatically confirmed PDEs in the *P. aeruginosa* genome, which may have partially overlapping functional outputs and whose sensory inputs remain obscure. Furthermore, the environment and nutrient availability (i.e., media used), modulates the phenotype of deletion strains (52, 53). What is striking from our results is how difficult it was to abolish the dispersal phenotype in a model with nutritionally rich but iron-poor media. The most parsimonious explanation is that at least some of these proteins can function interchangeably in response to a single signal, though we cannot exclude that there are three distinct signals originating from cyanide exposure that use each protein independently. RbdA, DipA and BifA are all well established as enzymatically active PDEs that generally regulate motility and dispersal, depending on the specific system (36, 40, 54). Additionally, DipA and BifA are conserved across all Pseudomonads (55). All three proteins have a generally polar colocalization, and, at least in the case of DipA, they function as part of a chemotactic molecular machine with the flagella,andincluding the chemosensory proteins (56). Feng et al. recently showed that the PDEs BifA, RbdA, DipA, along with another PDE, ProE, are all able to functionally complement each other in a Congo Red assay when constitutively expressed at low levels, reflecting possible functional redundancy at the PDE level in signaling (56). Additionally, Xin et al. recently showed that DipA and RbdA (as well as NbdA) each inhibit flagellar switching, again revealing potentially overlapping roles for these proteins (57). In the model proposed by Xin et al., each PDE is paired with a specific methyl-accepting chemotaxis protein complex and regulates a local pool of cyclic-di-GMP, with DipA and RbdA both controlling larger/deeper pools than NbdA. Our data are in general agreement with the above findings as cyanide dispersal does require cyclic-di-GMP signaling; however, at least under the current environmental conditions, there is redundancy in the requirement for specific PDEs.

In summary, we have found that abrupt inhibition of terminal oxidase activity leads to biofilm dispersal. In models resembling the host respiratory environment, multiple PDEs serve apparently redundant roles. Further work might focus on determining the specific link(s) between respiration and cyclic-di-GMP signaling, or the development of bacterial-specific respiratory inhibitors that could be exploited to promote biofilm dispersal.

## SUPPLEMENTAL MATERIAL

Supplemental Methods aem.01101-23-s0001.pdf Methods for screen.

Figures S1 to S3 aem.01101-23-s0002.pdf Supplemental Figures, legends included in file.

Table S1 aem.01101-23-s0003.pdf Bacterial strains and primers used.

Table S2 aem.01101-23-s0004.pdf PDE/DGC library screen results.

ASM does not own the copyrights to Supplemental Material that may be linked to, or accessed through, an article. The authors have granted ASM a non-exclusive, world-wide license to publish the Supplemental Material files. Please contact the corresponding author directly for reuse.
