# Supplementary material for: Bacterial respiratory inhibition triggers dispersal of Pseudomonas aeruginosa biofilms
Source: Appl Environ Microbiol. 2023 Sep 20;89(10):e01101-23. doi: 10.1128/aem.01101-23 (PMC10617509; doi:10.1128/aem.01101-23)
Supplement: Supplemental Methods — Methods for screen. [file aem.01101-23-s0001.pdf]

Screen description:

Experiments were done on 12-mm filters seeded with CFBE 41o- AECs, with 12 filters per plate. Each day we included PA14 parental control and KCN treat (one well each) to ensure the control PA14 well did not spontaneously disperse and that the KCN treated parental well did disperse. This left generally space to screen 10 strains. PAO1 has been much more extensively used in this model and has a much more reliable and robust dispersal phenotype; however the library we had available was made in PA14 – which decreased the dynamic range of the screen.

For the first screen, only the number dispersed with KCN were counted (recorded as CFU at 10-5 dilution, plated in duplicate and averaged (column C)

We then took the 8 strains with the fewest dispersed cells and repeated the dispersal experiment including a control well for each genotype (columns D and E).

Of these 8, we saw at least 2-fold increase in dispersed CFU for 6 strains and did not pursue the further. The remaining 2 strains were repeated a third time (55310, 45930) – and 45930 was the only strain that did not appear to disperse more than 2x across the multiple attempts.

There were two strains (deletions of PA14_07500 and PA14_60870 *morA*) that do not form adequate biofilm in the assay to feel comfortable excluding/including them in further assays in the model.
