# Supplementary material for: Bacterial respiratory inhibition triggers dispersal of Pseudomonas aeruginosa biofilms
Source: Appl Environ Microbiol. 2023 Sep 20;89(10):e01101-23. doi: 10.1128/aem.01101-23 (PMC10617509; doi:10.1128/aem.01101-23)
Supplement: Figures S1 to S3 — Supplemental Figures, legends included in file. [file aem.01101-23-s0002.pdf]

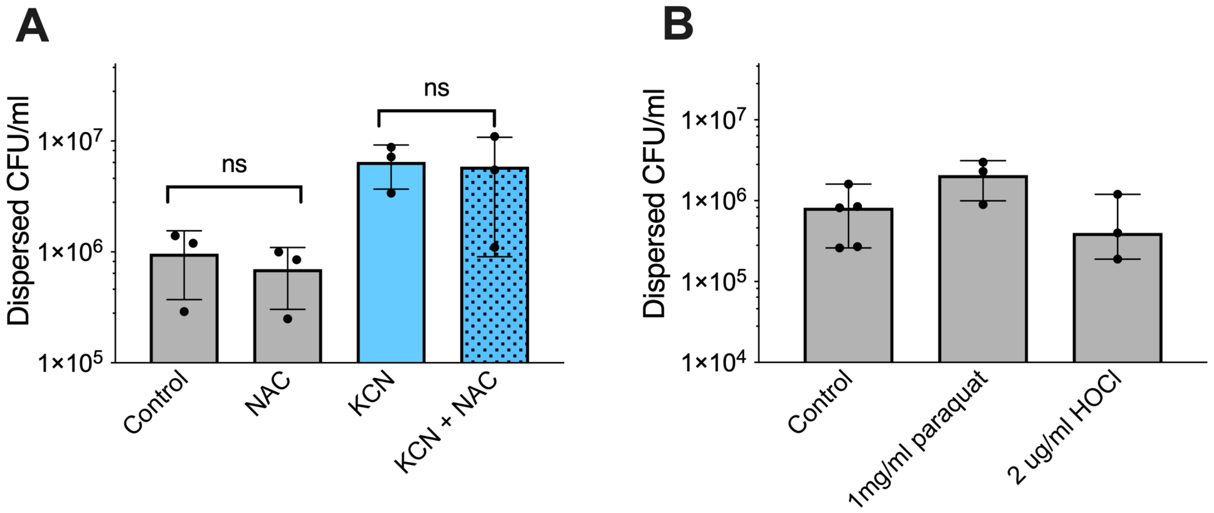

Figure S1: Oxidant exposure does not trigger dispersal. PAO1 biofilms were grown on the surface of AECs for 6 hours and dispersed with 300 μM KCN. The addition of 10 mM N-acetyl cysteine (NAC) did not block dispersal. (B) Treatment of biofilms grown on AECs with paraquat and sodium hypochlorite did not trigger biofilm dispersal. One-way ANOVA was done followed by pair-wise testing, indicated comparisons had a p-value >0.05.

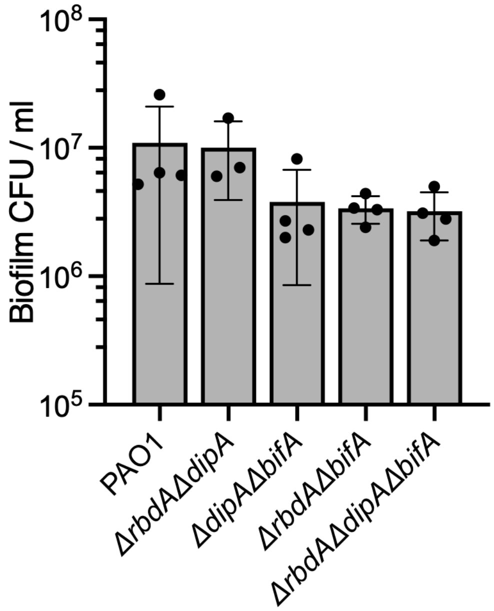

Figure S2: Biotic biofilm formation of deletion strains at six hours. Biofilms of the indicated strains were grown on AECs and quantified by serial dilution. As measured by CFUs, biofilm formation was similar between strains.

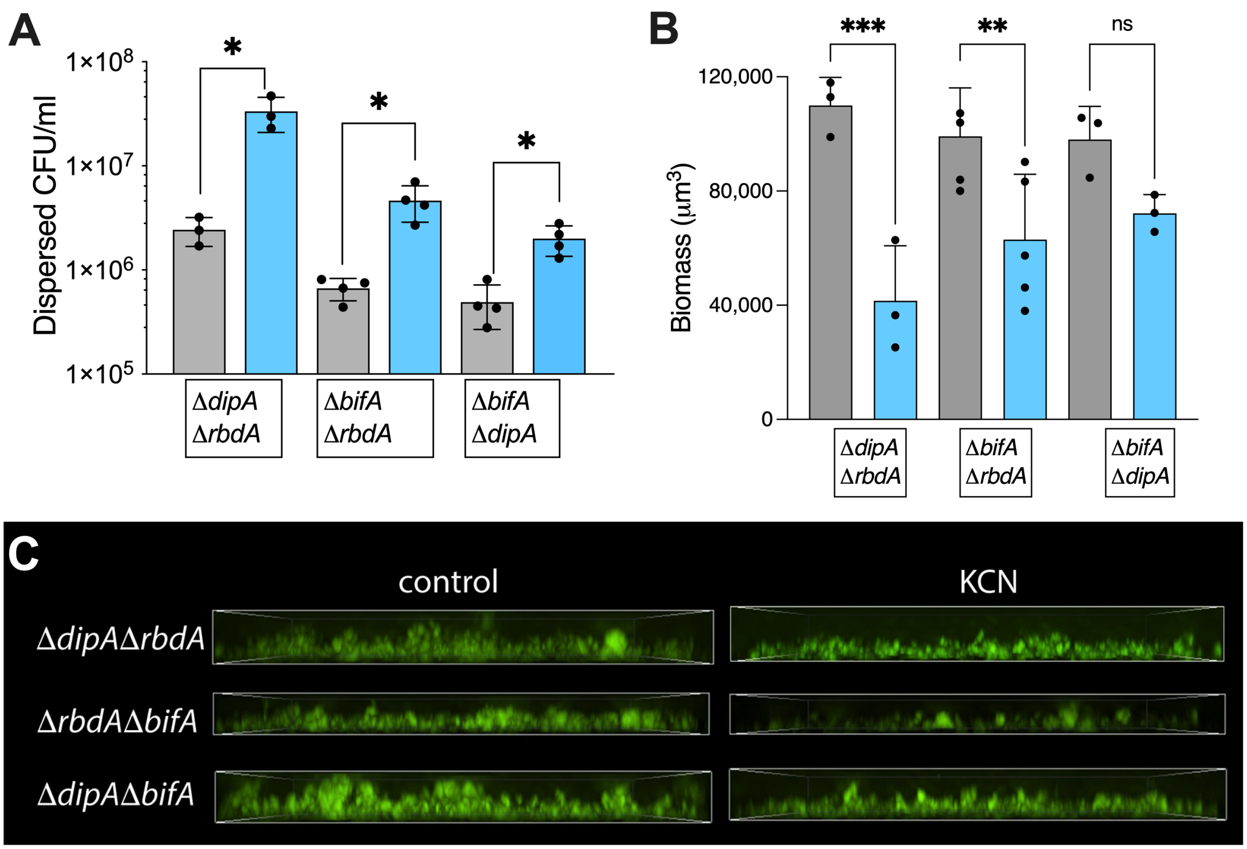

Figure S3: Dispersal of PDE combined deletion strains. (A) Biofilms of double deletion strains were grown on CFBE41o- airway epithelial cells and dispersed with 300 μM KCN for 15 minutes. Dispersed bacteria were counted by serial dilution. (B-C) Biofilms of indicated strains were grown on glass in MEM-Fe for 6 hrs. (B) Biomass quantified with representative maximum intensity projection x-z stacks shown in (C). * indicates significance level by one-way ANOVA followed by Sidak's multiple comparison test.
