# Supplementary material for: Bacterial respiratory inhibition triggers dispersal of Pseudomonas aeruginosa biofilms
Source: Appl Environ Microbiol. 2023 Sep 20;89(10):e01101-23. doi: 10.1128/aem.01101-23 (PMC10617509; doi:10.1128/aem.01101-23)
Supplement: Table S1 — Bacterial strains and primers used. [file aem.01101-23-s0003.pdf]

Supplemental Table 1: Bacterial Strains and Primers Used

***P. aeruginosa* Strain**	**Description**	**Source**
PAO1	Parental strain	G. O'Toole
PA14	Parental strain	G. O'Toole
PAO1-gfp	PAO1 with constitutive gfp plasmid	G. O'Toole
Δ*bifA*	In-frame deletion of *bifA*	(2)
Δ*dipA*	In-frame deletion of *dipA*	(2)
Δ*rbdA*	In-frame deletion of *rbdA*	(2)
Δ*rbdA*Δ*dipA*	In frame deletion of *rbdA* and *dipA*	This paper
Δ*rbdA*Δ*bifA*	In frame deletion of *rbdA* and *bifA*	This paper
Δ*dipAA*Δ*bifA*	In frame deletion of *dipA* and *bifA*	This paper
Δ*rbdA*Δ*bifA*Δ*nbdA*	In frame deletion of *rbdA, bifA* and *dipA*	This paper
PAO1 - Δ*lapGD*	In frame deletion of *lapGD,*	This paper
PAO1 – Δ*lapGD::p*UC18-miniTn7-*lapGD*	Complementation strain using the predicted endogenous promoter.	This paper
PA14 - Δ*lapD*	In frame deletion of *lapD*	(3)
Δ*cioAB*	In frame deletion of *cioAB*	This paper
PAO1::pUC18-miniTn7-p_{ntp2}-*cioAB*	Constitutive expression of *cioAB*	This paper
**Plasmids**	**Description**	**Reference**
pMQ30	Broad host allelic recombination vector, used for cioAB deletion	(4)
pTNS3		(5)
pUC18-mini-Tn7-tet-pntp2	Mini-Tn7 targeting plasmid, for cioAB constituative expression	(6)
pEX18-Gm	Gateway plasmid, used for lapGD deletion	(7)
pMQ30-dipA plasmid	Allelic recombination plasmid for deletion of *dipA*	(2)
pEX18-bifA plasmid	Allelic recombination plasmid for deletion of bif*A*	(2)
pMQ30 - rbdA plasmid	Allelic recombination plasmid for deletion of rbd*A*	(2)
**Primers**	**Sequence**	**Description**
cioUpF	gagaattcgcctttgttgaagttctccagg	Deletion strain construction
cioUpR	cctcctcagtgatagccatcgggcaactcctcttcaggttctc	Deletion strain construction
cioDownF	gagaacctgaagaggagttgcccgatggctatcactgaggagg	Deletion strain construction
cioDownR	cagaattccagatgagcttccatgagtggc	Deletion strain construction
lapGDUpF	ggggacaagtttgtacaaaaaagcaggcta catcgttggccttcagggt	Deletion strain construction – using GW
lapGDUpR	cgctccggactcgctaatcgagcgggctcc	Deletion strain construction
lapGDDownF	ggagcccgctcgattagcgagtccggagcg	Deletion strain construction
LapGDDownR	ggggaccactttgtacaagaaagctgggtatgttcaaggagcgcaaagg	Deletion strain construction – using GW
cioAB_Fwd	TCCTCATCCTGTCTCTTG	Complementation, NEB Builder System
cioAB_Rev	CAACCAGATAAGTGAAATCTAG	Complementation, NEB Builder

		System Complementation
Pntp2Tet_Fwd	atcaagagacaggatgaggaATGTTCGGATTGGAGGCTATC	Complementation, NEB Builder System
Pntp2Tet_rev	gatttcacttatctggttggTCAGTGATAGCCATCGCC	Complementation, NEB Builder System
45930_OperonFwd	gtcaACTAGTACCATCTGCCCTTCGCTC	Complementation, includes SpeI digestion site
45930_OperonRev	gtcaAAGCTTACTCGCTATCAGGCATCC	Complementation, includes HindIII digestion site

Additional Notes:
The homologous recombination plasmid pEX18-Gm was used for the deletion of Bif. pMQ30 was used for deletion of cioAB, RbdA and DipA.

- Melvin JA, Gaston JR, Phillips SN, Springer MJ, Marshall CW, Shanks RMQ, Bomberger JM. 2017. Pseudomonas aeruginosa Contact-Dependent Growth Inhibition Plays Dual Role in Host-Pathogen Interactions. mSphere2017/11/21. 2.
- Zemke AC, D'Amico EJ, Snell EC, Torres AM, Kasturiarachi N, Bomberger JM. 2020. Dispersal of Epithelium-Associated Pseudomonas aeruginosa Biofilms. mSphere 5.
- Ha DG, Richman ME, O'Toole GA. 2014. Deletion mutant library for investigation of functional outputs of cyclic diguanylate metabolism in Pseudomonas aeruginosa PA14. Appl Environ Microbiol2014/03/25. 80:3384–3393.
- Shanks RM, Caiazza NC, Hinsa SM, Toutain CM, O'Toole GA. 2006. Saccharomyces cerevisiae-based molecular tool kit for manipulation of genes from gram-negative bacteria. Appl Environ Microbiol2006/07/06. 72:5027–5036.
- Choi KH, Mima T, Casart Y, Rholl D, Kumar A, Beacham IR, Schweizer HP. 2008. Genetic tools for select-agent-compliant manipulation of Burkholderia pseudomallei. Applied and Environmental Microbiology https://doi.org/10.1128/AEM.02430-07.
- Torres A, Kasturiarachi N, DuPont M, Cooper VS, Bomberger J, Zemke A. 2019. NADH dehydrogenases in Pseudomonas aeruginosa growth and virulence. Frontiers in Microbiology 10.
- TT. H, RR. K-S, AJ. K, HP. S. 1998. A broad-host-range Flp-FRT recombination system for site-specific excision of chromosomally-located DNA sequences: application for isolation of unmarked Pseudomonas aeruginosa mutants. Gene 212.
