# Supplementary material for: Bacterial respiratory inhibition triggers dispersal of Pseudomonas aeruginosa biofilms
Source: Appl Environ Microbiol. 2023 Sep 20;89(10):e01101-23. doi: 10.1128/aem.01101-23 (PMC10617509; doi:10.1128/aem.01101-23)
Supplement: Table S2 — PDE/DGC library screen results. [file aem.01101-23-s0004.pdf]

PA_14 locus	Gene Name	Replicate 1 KCN	Replicate 2 Control	Replicate 2 KCN	Replicate 3 Control	Replicate 3 KCN	Was dispersal seen?
53310		12.3	8.3	13	4.00E+04	1.50E+05	yes
26970		13	3	15			yes
45930	lapD	13	13	17	5.90E+04	8.00E+04	see Figure 3
4420		12	5	20			yes
2110	siaD	12	8	19			yes
69900		13	7	24			yes
42220	mucR	13	8.3	21			yes
16500	wspR	13	10.7	25			yes
**Strains that were not rescreened**							
21190	nbdA	14					
65090	nicD	14					
20820		14					
57140		14					
56790	bifA	14	See Figure 4				
3790		14.7					
23130		15					
30830		16					
49890	yfiN	16					
59790	pvrR	17					
40570		17					
64050	gcbA	17					
36260		17.6					
36990		18					
63210		19					
31330		19					
56280	sadC	20					
37690		22					
50060	roeA	24					
72420		25					
21870		27					

14530		27	
71850		*27*	
10820		28	
3720		30	
49160		34	
66320	dipA	34	See Figure 4
65540	fimX	40	
12810	rocR	41	
53140	rbdA	49	See Figure 4

Inadequate Biofilm formation for analysis

7500		1-log lower biofilm formation
60870	morA	1-log lower biofilm formation

These data are the pooled control wells for each day of the screen.

Control CFU dispersed	300 uM KCN CFU dispersed	Log Control CFU	Log KCN CFU
8.00E+04	1.73E+05	4.9	5.2
6.60E+04	2.50E+05	4.8	5.4
1.40E+05	4.70E+05	5.1	5.7
5.00E+04	1.00E+05	4.7	5.0
9.30E+04	1.30E+05	5.0	5.1
5.30E+04	1.60E+05	4.7	5.2
4.30E+04	3.60E+05	4.6	5.6
5.60E+04	2.40E+05	4.7	5.4
**Mean**	**Mean**		**p-value, t-test**
**7.26E+04**	**2.35E+05**		**0.00027597**

Table S2: PDE/DGC Library screen results. The locus number and strain name (if available) is shown. The raw number of dispersed CFU (x10^4) bacteria for each replicate. If loss of dispersal from the first replicate was suspected based on the KCN dispersal CFU, then a second replicate was done with both control and KCN data shown. Equivocal results were seen for two strains and a third replicate was done. At the bottom of the table are shown the pooled values for the control (parental) strain for each experimental day.
